# Microneedle-enhanced drug delivery: fabrication, characterization, and insights into release and permeation of nanocrystalline imiquimod

**DOI:** 10.3389/fddev.2024.1425144

**Published:** 2024-06-27

**Authors:** Sophie Luise Meiser, Jonas Pielenhofer, Ann-Kathrin Hartmann, Lara Stein, Jule Dettweiler, Stephan Grabbe, Markus P. Radsak, Peter Langguth

**Affiliations:** ^1^ Department of Biopharmaceutics and Pharmaceutical Technology, Johannes Gutenberg University Mainz, Mainz, Germany; ^2^ 3rd Department Internal Medicine, University Medical Center, Johannes Gutenberg University Mainz, Mainz, Germany; ^3^ Institute of Immunology, University Medical Center of the Johannes Gutenberg-University Mainz, Mainz, Germany; ^4^ Department of Dermatology, University Medical Center, Johannes Gutenberg University Mainz, Mainz, Germany

**Keywords:** microneedles, dissolvable microneedle, transdermal delivery, *Ex vivo* permeation, imiquimod

## Abstract

Transcutaneous delivery systems bear several advantages over conventional needle-based injections. Considering the low bioavailability and poor water-solubility of imiquimod, a manufacturing process has been developed to incorporate imiquimod as suspended nanocrystals in different formulations. In this study, three formulations - fast-dissolving microneedle arrays that contain nanocrystalline imiquimod in a poly (vinyl)alcohol matrix and two semisolid preparations-were characterized and compared. The results show that microneedle arrays have an advantage over the semisolid preparations regarding *in vitro* release and permeation characteristics. Microneedle arrays facilitate *ex vivo* permeation, thus reducing the applied dose by 93% compared to the semisolid formulations. Additionally, the amount of imiquimod permeated after 24 h maintained the same level even when the contact time of the formulation with the skin is less than 1 hour. In conclusion, our results highlight the great potential of advanced microneedle based delivery systems and foster the further evaluation of this approach.

## 1 Introduction

Microneedle arrays (MNA) are a popular subject of research for developing formulations for transdermal drug delivery due to their high potential for clinical use (Prausnitz, 2004; Leone et al., 2017; Kirkby et al., 2020; Phatale et al., 2022). Dermal drug application with MNAs was first described in the late 1990s (Henry et al., 1998), and since then, the number of publications on the topic has been growing every year. Major areas of research include manufacturing processes (Prausnitz, 2017; Henriquez et al., 2023), (polymer) matrices (Donnelly et al., 2011; Ahmed Saeed Al-Japairai et al., 2020), and strategies for embedding potential drugs (Park et al., 2006; Dillon et al., 2019). MNAs can be classified into three types: solid (possibly coated or porous), hollow, as well as biodegradable, and/or dissolving (Donnelly et al., 2012). This study focuses on fast-dissolving MNAs made of Poly (vinyl)alcohol, which has been used as a matrix polymer in previous studies (Nguyen et al., 2018). The advantages of this particular approach include the sharpness of the microneedles, which allows for painless penetration of the stratum corneum without generating harmful or infectious waste. Moreover, MNAs are cost-effective and user-friendly tools, even for individuals without medical training (Prausnitz et al., 2009; Alkilani et al., 2015; Ita, 2015; Larrañeta et al., 2016; González-Vázquez et al., 2017).

Considering the various concepts exploring the skin as a target site and the abundant presence of immune cells, particularly Langerhans cells and dermal dendritic cells (Engelke et al., 2015; Kaurav et al., 2016), the skin appears to be a promising destination for drug delivery through dermal formulations, especially in the context of transcutaneous immunization (Karande and Mitragotri, 2010; Donnelly et al., 2012; Ita, 2014; Leone et al., 2017; Hirobe et al., 2022). Our previous research has already focused on this area (Sabri et al., 2020), and we aim to further investigate this topic.

Imiquimod (IMQ) is a US Food and Drug Administration (FDA)-approved drug used as an active pharmaceutical ingredient (API) in the investigated formulations for the treatment of actinic keratosis, superficial basal cell carcinomas, and anogenital warts. IMQ’s main mechanisms of action involve its agonistic activity towards toll-like receptors (TLR-7 and TLR-8), which induce proinflammatory cytokines (Megyeri et al., 1995; Schön and Schön, 2007; Hanna et al., 2016). Regarding the mode of action of IMQ, particularly in the skin and through topical administration of the API, it was found to have potential as an adjuvant for transcutaneous immunization (TCI) (Warger et al., 2007; Hung et al., 2016; Stein and Radsak, 2016; Lopez et al., 2017). However, the commercially available product for dermal application of IMQ “Aldara™” did not demonstrate sufficient memory formation in previous studies (Warger et al., 2007). Therefore, the formulation was further improved, transitioning from a semisolid formulation to the development of IMQ-loaded MNA.

Incorporating suspended drugs in a dissolvable matrix is an elegant solution for formulating treatments because it increases the physical stability of the disperse system while enabling a rapid drug release. The use of nanocrystalline drugs to improve their bioavailability (Chogale et al., 2016), poses challenges for formulation in terms of achieving adequate homogeneity and scalability. However, this approach also offers potential benefits. Mixing fluids of different viscosities can be particularly challenging, especially when using MNA tips that can only hold very low volumes. To address this challenge, we developed a gentle and scalable mixing procedure that ensures sterility throughout the manufacturing process of the MNA.

Previous studies have described the advantages of formulating drugs at the nanoscale, particularly in delivering medication trans- and/or intradermally (Prausnitz and Langer, 2008; Prow et al., 2011; Patzelt and Lademann, 2020; Pielenhofer et al., 2020). Our research group has built upon these insights by developing, enhancing, and characterizing semisolid formulations containing nanocrystalline IMQ (Gogoll et al., 2016; Pielenhofer et al., 2023). One of the resulting formulations, IMIGel, is currently being used as an investigational medicinal product (IMP) in an academic phase-I/II clinical trial (EudraCT: 2015-002203-28). The other formulation, IMISol+, involves different oil components and includes a freeze-drying step in the manufacturing process.

In our effort to improve these formulations, we explored the use of MNA as an alternative platform. This required conducting comparative tests, such as *ex vivo* permeation studies of the active pharmaceutical ingredient (API). This study demonstrates a suitable manufacturing process for formulating nanocrystalline IMQ in MNAs and the advantages of MNAs over the examined semisolid formulations. Additionally, the dose equivalence for our formulations based on these investigations has been determined. This finding will be valuable in evaluating future investigations, building upon the groundwork laid by previous research (Lopez et al., 2017; Sohl et al., 2022).

## 2 Materials and methods

### 2.1 Materials

EMPROVE^®^ Essential, poly (vinyl alcohol) 18–88 was kindly provided by Merck KGaA, Darmstadt, Germany. Imiquimod (IMQ) drug substance of GMP grade was obtained from Teva Pharmaceutical Industries Ltd.–API Division (Debrecen, Hungary). Sucrose fatty acid ester D-1816 was kindly provided by Harke Pharma (Mitsubishi-chemical food corporation (Tokyo, Japan)). MNA templates ST 10 × 10 H600B200P500 were purchased from Micropoint Technologies Pte, Ltd., CleanTech Loop (Singapore). Static mixer (cartridges and mixing elements) AB 025–01-10–01 Set-B 25, 1:1/AB 050–02-10–33 Set-B 50, 2:1; MBH 05-20T/MBH 06-20T were purchased from Adchem GmbH (Wendelstein, Germany). Aponorm 7 mL tubes, Aqua conservata DAC, Carbopol 974P, jojoba wax, polysorbate 80, isopropylmyristate, medium chain triglycerides, and sodium hydroxide were purchased from Caesar & Loretz GmbH (Hillscheid, Germany). Zirconium oxide milling spheres of 1 mm diameter were purchased from Fritsch GmbH (Idar-Oberstein, Germany). Poloxamer 407 (Kolliphor P407) was kindly provided by BASF (Ludwigshafen, Germany). Acetonitrile ≥99.9%, HiPerSolv CHROMANORM gradient grade and methanol ≥99.8%, HiPerSolv CHROMANORM gradient grade, WTW technical buffers TEP 2, STP-7, squalene, tocopherol, aerosol, acidic acid, sodium acetate and STP-10 trace were purchased from VWR International GmbH (Darmstadt, Germany). Phosphoric acid LiChrompur >85%, PBS buffer pH 7.4, sulfuric acid ≥98% LiChropur, and triethylamine ≥99.5% Li-Chropur were purchased from Sigma Aldrich Chemie GmbH (Taufkirchen, Germany). Heptane-1-sulphonic acid sodium salt, and OMNAifix disposable 50 mL syringes with Luer-Lock fitting were purchased from Carl Roth GmbH (Karlsruhe, Germany). Inertsil ODS3-5 µm 250 mm × 4.6 mm and Zorbax RX-C8 150 mm × 4.6 mm 5 µm HPLC columns were purchased from MZ Analysentechnik GmbH (Mainz, Germany). Porcine cadaver ear skin was utilized as samples with full skin thickness. The porcine ears were sourced from a nearby slaughterhouse and stored after dissection at −21°C until usage.

### 2.2 Manufacturing of nanocrystalline IMQ suspension

#### 2.2.1 Milling of imiquimod nanocrystals

To create a nanocrystalline suspension of IMQ, a top-down approach utilizing wet media ball milling was employed, targeting a particle size with a z-average of 400 ± 200 nm. The milling process was slightly modified for each formulation to achieve comparable particle sizes while altering the composition.

For the MNAs, 3.0 g of imiquimod drug substance was combined with 17 g of a 0.9% (w/w) solution of polysorbate 80 in MilliQ water, along with 25 g of 0.5 mm zirconium oxide grinding spheres in a 45 mL zirconium oxide grinding vessel. The mixture was milled for 140 min, divided into five 20-min cycles with a 10-min pause interval between each cycle, using a planetary ball mill (Fritsch Pulverisette 6). Subsequently, the suspension was filtered using suction to remove the milling balls from the formulation.

The details of the milling process setup for manufacturing nanocrystalline IMQ suspensions for the semisolid formulations will be provided in Section 2.5.

#### 2.2.2 Mixing of the molding compound

The matrix polymer used for the MNA spikes was poly (vinyl)alcohol (PVA 18-88) at a concentration of 15% (w/w). To create the molding compound, the nanocrystalline suspension of IMQ (described in 2.2.1) and the matrix polymer were mixed in a ratio of 2:1 using a set of static mixers.

### 2.3 Manufacturing of the microneedle arrays

Microneedles were produced using the solvent cast method, which has been previously described (Kim et al., 2012; Abdelghany et al., 2019). To prepare the spikes of the MNA, a 2:1 mixture of polymer (15% (w/w) PVA 18–88 in MilliQ) and imiquimod suspension (40 mg) (described in 2.2.2.) were applied to silicone templates (Micropoint, 600 μm, 10 × 10 MNA of pyramidal shape). The templates were then centrifuged using an Eppendorf centrifuge 5810 R (3,200 rpm, 10 min), after which the supernatant was removed, and the templates were left to incubate at 37°C for 24 h.

To create the backing layer of the MN arrays, PVP (K 30 1 g/mL in MilliQ) was applied to the templates, centrifuged again, and applied once more to fill the entire mold. The templates were then allowed to harden by being stored in a desiccator at room temperature for 48 h before being removed from the templates. The completed MN were stored in the desiccator at room temperature until ready for use.

### 2.4 Characterization of nanocrystalline IMQ suspension

#### 2.4.1 Particle size analysis of nanocrystalline imiquimod suspension

Intensity-weighted particle size was determined using a Malvern Zetasizer Nano ZS ZEN 3600. The diluted suspension was placed in a disposable cuvette and the particle size was measured.

In previous studies of our group, the nanocrystalline structure of the milled IMQ particles was also examined using transmission electron microscopy (Stein et al., 2014), which will not be part of this study.

#### 2.4.2 Mixing quality of molding compound

To ensure homogenous drug loading, the mixing quality of the molding compound was analyzed using High-Performance Liquid Chromatography (HPLC) with UV detection on a Jasco HPLC system (LC-Net II/ADC interface box, Autosampler AS-950, PU-980 pump, UV-VIS UV-975 detector, DG980-50 degasser, LG-980–50 gradient mixer, Jet Stream ATP-CHY 501 column oven). Chromnav CFR 2 software was used to analyze the resulting peaks. The USP Monograph Imiquimod Cream Assay method (The United States Pharmacopeia and National Formulary, 2018) was used for analysis, with slight modifications in sample preparation.

### 2.5 Characterization of IMQ-loaded microneedle arrays

#### 2.5.1 Optical appearance

The MNAs, both with and without IMQ loaded into the spikes, were visually assessed using a Zeiss Discovery V12 microscope. Only those MNAs that were optically intact and uniform were selected for further experiments.

#### 2.5.2 Insertion success

To evaluate the successful insertion of the MNA, incubated porcine cadaver skin was treated with both placebo and drug-loaded MNA by administering them with a defined force of 9 N. The MNAs were removed after 30 s and the skin was stained with methylene blue solution for 20 min. The remaining microlesions were then counted by using a Zeiss Discovery V12 stereomicroscope.

Additionally, the transepidermal water loss (TEWL) was measured before and after insertion using a Tewameter^®^ TM 300 w to examine the extent of epidermal perforation, as previously described (Gomaa et al., 2010).

#### 2.5.3 Fraction force

To ensure the stability of the MNA during application, the fraction force of the backing layer was characterized by using a TA. XT plus Texture Analyzer from Stable Micro Systems. To determine the maximum force applied to the backing layer of a complete MNA before breakage, a three-point bend test was selected. The test was conducted with a test speed of 2 mm/s, with a trigger force set to 0.049 N.

#### 2.5.4 Drug loading of microneedles

To determine the amount of IMQ loaded to the MNA, the USP Imiquimod Cream Assay was performed using HPLC (comparable to analysis in 2.3.2.). For sample preparation, one MNA was fully dissolved in 12.5 mL of diluent.

### 2.6 Manufacturing of semisolid nanocrystalline IMQ formulations

#### 2.6.1 Manufacturing of IMIGel

**FIGURE 1:**
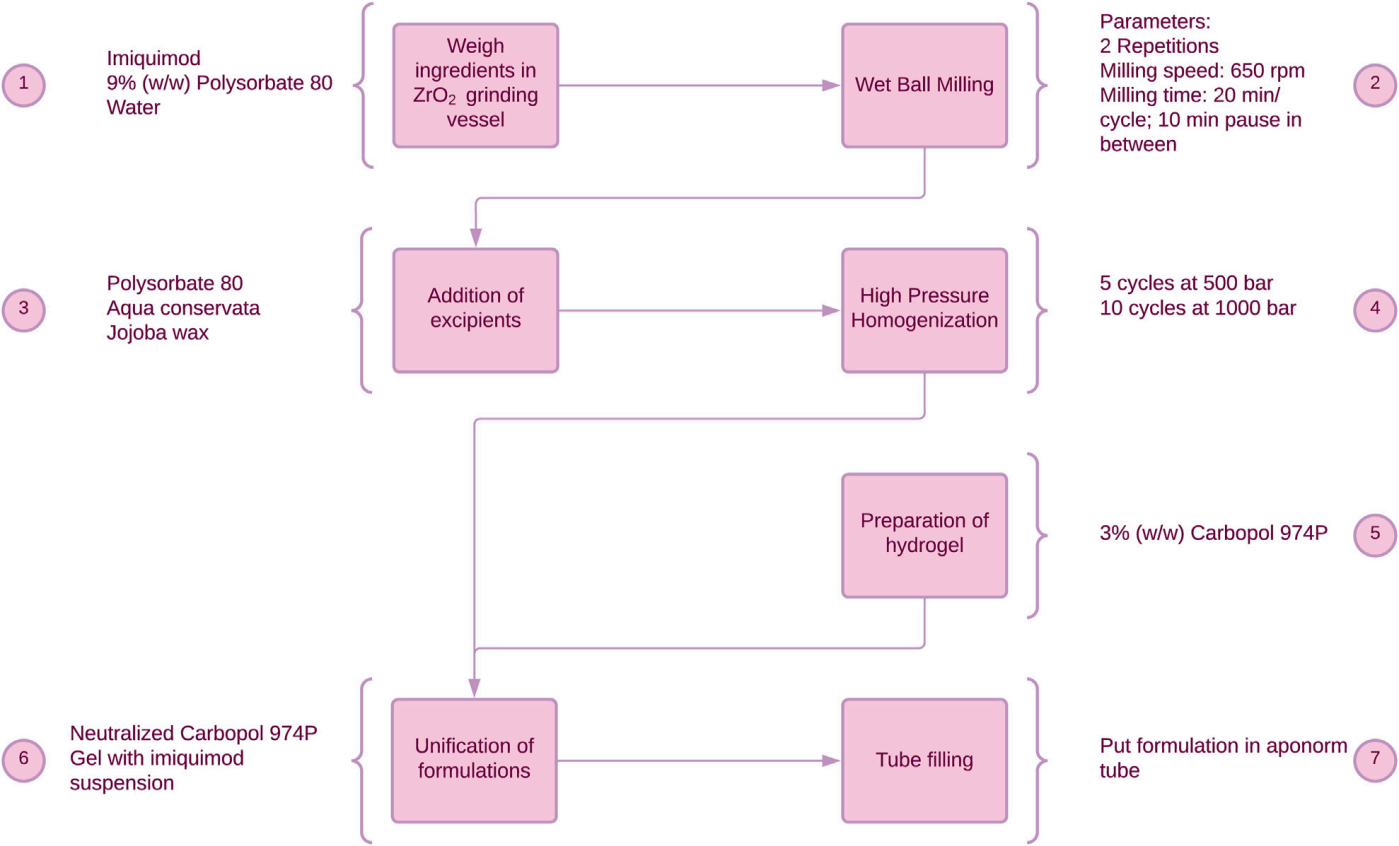
Manufacturing process of IMIGel. IMQ is being processed to nanocrystalline dimensions in the milling process (see step 2).

IMIGel as a semisolid nanocrystalline formulation of IMQ was manufactured and used as an investigational medicinal product (IMP) in an academic phase-I/II clinical trial (EudraCT: 2015–002203-28). The composition and manufacturing procedures were thoroughly illustrated in previous research (Pielenhofer et al., 2023). In brief, the processing steps are depicted in Figure 1, providing a concise overview of the manufacturing procedures.

#### 2.6.2 Manufacturing of IMISol+

**FIGURE 2:**
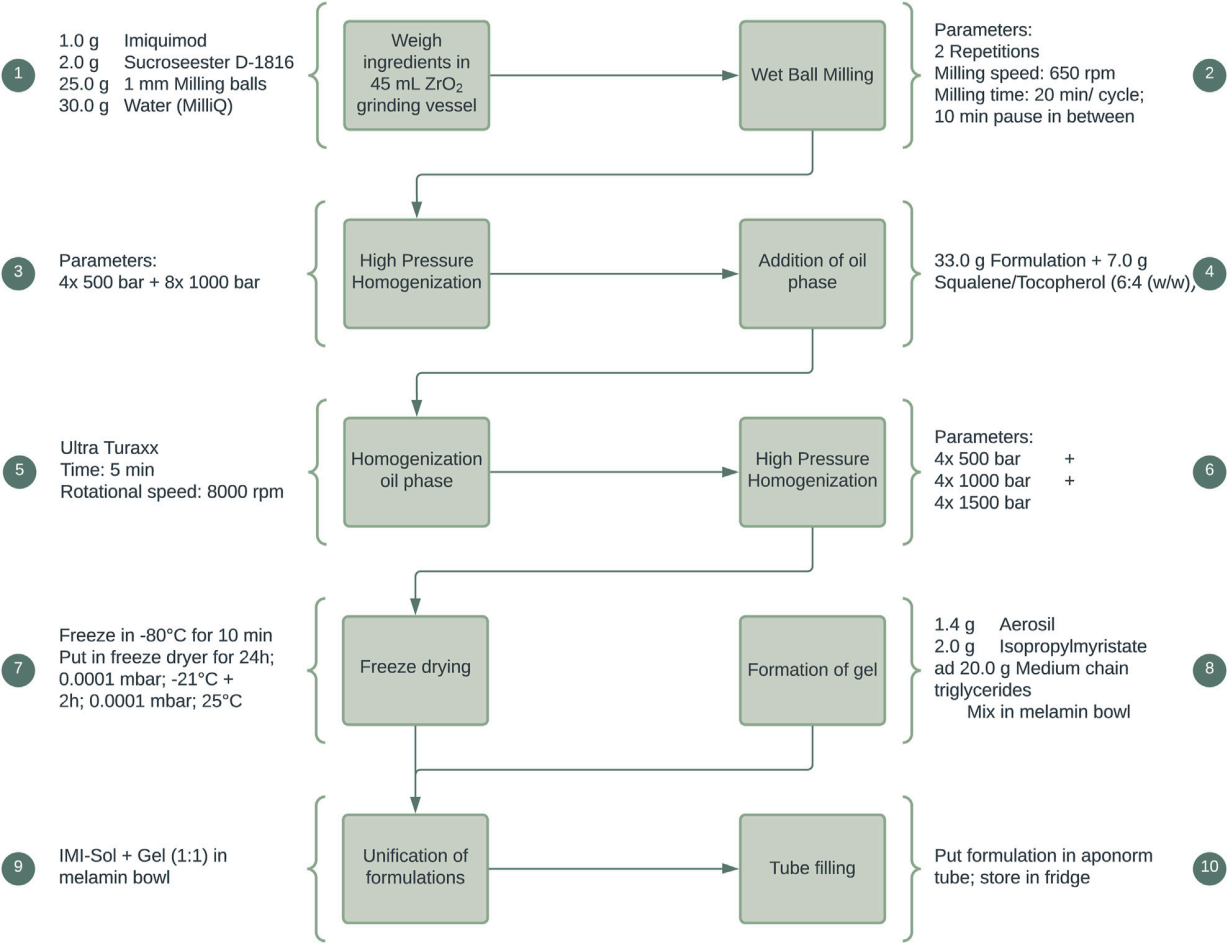
Manufacturing process of the semisolid formulation IMISol+.

As a further progression, a solid nanoemulsion of IMQ, IMISol, was developed. This formulation incorporated several modifications, including replacing the previous oil phase consisting of jojoba wax with a mixture of squalene and tocopherol in a ratio of 6:4 (w/w). Additionally, a freeze-drying step was introduced, as illustrated in Figure 2. The development, the manufacturing process and special properties of the formulation have been described in previous work (Gogoll et al., 2016). To ease the application the two process steps 7 + 8 (Figure 2) were subsequently added, resulting in the formulation IMISol+, which was already used for further investigations (Hartmann et al., 2023). This formulation comprises the redispersion of the nanoemulsion containing nanocrystalline IMQ, along with an oleogel composed of aerosil, isopropyl myristate, and medium-chain triglycerides.

### 2.7 *In vitro* release of nanocrystalline IMQ comparing dissolvable MNA to semisolid formulations

To compare the semisolid formulations and the MNA towards their *in vitro* release profile, the samples were placed in 7.5 mL acetate buffer (pH 3.7, 100 mM) at 32°C including a magnetic stirrer (700 rpm). The MNAs were attached to a sinker, and the semisolid formulations (20 mg) were filled into one-side opened glass vials. Samples of 400 µL were taken at 0, 10, 30, 60, 120, 180, 240, and 1,440 min and replaced with tempered buffer. To determine the amount of IMQ in the samples, the USP Imiquimod Cream Assay was performed using HPLC (comparable to analysis in 2.3.2.).

### 2.8 *Ex vivo* permeation of nanocrystalline IMQ comparing dissolvable MNA to semisolid formulations

To investigate and compare the passive *ex vivo* diffusion of nanocrystalline IMQ in different formulations, Franz diffusion cells (EDC-07 Franz-diffusion-cell apparatus, Labswiss, Muttenz, Switzerland) respectively vertical diffusion cell (VDC) were utilized. Full-thickness porcine cadaver ear skin was chosen as the diffusion membrane, as it has been identified as a suitable model for human skin (Jacobi et al., 2007). VDCs with a receptor volume of 7.5 mL (excluding stirrer) were filled with degassed acetate buffer pH 3.7 (100 mM) (Donnelly et al., 2006) and maintained at a temperature of 32°C ± 1°C, following the USP specifications for semisolid drug products performance testing general chapter 〈1724〉 (The United States Pharmacopeia and National Formulary, 2018). Water-jacketed receptor cells connected to a thermostatically controlled water bath were employed to ensure temperature equilibration. Pretest equilibration was carried out for 30 min. After applying the membranes to the vials, magnetic stirrers were set at a speed of 700 rpm. The various formulations (IMINeedle, IMIGel, IMISol+) were applied to the skin samples. Duplicate measurements were conducted in each of the three experimental runs to ensure reliability. A line-up of the applied samples and the corresponding doses of IMQ is listed in Table 1. Sampling was carried out at specific time intervals, including 0, 10, 30, 60, 120, 180, 240, and 1,440 min. At each interval, 0.7 mL of receptor medium samples were simultaneously withdrawn from each VDC. To maintain consistency, the withdrawn sample volume was promptly replaced with a fresh, temperature-equilibrated receptor medium.

**TABLE 1:** Detailed overview of the samples used for Franz diffusion cell testing, along with the corresponding doses of IMQ applied.

Formulation	Content IMQ	Sample size applied	Dose applied [µg]
IMINeedle	70 µg/MNA	1 MNA	70
IMIGel	5%	20 mg	1,000
IMISol+	5%	20 mg	1,000

Concentrations of IMQ in the samples were determined using HPLC (comparable to 2.3.2.). The cumulative amount of IMQ permeated through the membrane was calculated by analyzing the peak area.

### 2.9 *Ex vivo* comparison on different application durations

To investigate the effect on the *ex vivo* drug permeation caused by the contact-time of the formulations with the skin, we performed a Franz diffusion cell experiment (as described in 2.7) with modifications in the application of the formulation. Specifically, we varied the duration of application by detaching the MNA or removing the semisolid formulations from the porcine ear skin after 10, 30, and 1,440 min (24 h). Samples were collected and analyzed according to 2.7.

### 2.10 *In vivo* effect of IMQ in MNA

*In vivo* testing of the drug effect of IMQ was investigated using a murine model, in detail C57BL/6 mice. The experiments were reviewed and approved by the local authorities (Federal Investigation Office Rhineland-Palatinate, Koblenz, Germany, approval no. 23 177–07/G 22-1-097). As IMQ can induce psoriasis-like symptoms in murine models (Chuang et al., 2018), the effective administration of the MNA was confirmed by measuring the ear thickness and the increase in thickness of the viable epidermis in H&E-stained ear skin samples. Microneedles were manually applied to the mouse ears by thumb pressure for 30 s. The thickness of the total mouse ear was measured using a thickness gauge before and on day two after treatment administration. On day seven, the thickness of the viable epidermis was examined in H&E-stained ear skin sections using a Zeiss Discovery V12 stereomicroscope.

### 2.11 Statistical data analysis and generation of graphs

The process flow charts were generated using Lucidchart 2023. HPLC chromatograms and peak integration were performed using ChromNAV2 CFR software (version 2.02.08). Peak analysis was conducted using Microsoft Excel 2019. Graphs illustrating the mixing quality, fracture force, drug loading, and *ex vivo* permeation were plotted using GraphPad Prism 10. Descriptive statistics were calculated using Minitab 20.4 and GraphPad Prism 10. Statistical analysis of the Franz diffusion cell data was performed using a one-way ANOVA (*p* < 0.05) with Tukey’s *post hoc* test (*p* < 0.001). Data from *in vivo* testing were statistically analyzed by performing t-tests (*p* < 0.05).

## 3 Results

### 3.1 Characteristics of nanocrystalline IMQ suspension

#### 3.1.1 IMQ suspension for semisolid formulation

Despite the utilization of different surfactants to ensure adequate wettability in the milling process to produce a nanocrystalline IMQ suspension as an intermediate for IMIGel and IMISol+ (as described in Section 2.5), comparable particle sizes were achieved while maintaining the milling process parameters. The IMQ suspension intended for the production of IMIGel exhibited a mean particle size (z-average) of 379 ± 11.2 nm and a polydispersity index (PdI) of 0.195 ± 0.03, while the suspension for IMISol + displayed a mean particle size of 432 ± 6.7 nm and a PdI of 0.299 ± 0.01.

By ensuring a nanoscale particle size, our objective is to enhance the transdermal delivery of IMQ. By maintaining this parameter consistent across all formulations, the results of *in vitro, ex vivo,* and *in vivo* experiments can be more effectively compared. Additionally, monitoring the particle size of an intermediate is crucial for process control during manufacturing processes according to Good Manufacturing Practice (GMP) guidelines.

#### 3.1.2 IMQ suspension for MNA arrays

To ensure homogeneity in the final product, especially in the MNA, it is important to pay attention to the quality of the intermediate nanocrystalline IMQ suspension before the molding process. This study focuses on two key parameters:

• Particle size
• Mixing Quality

The target particle size of the suspension was selected to be comparable to that of the semisolid formulations discussed in section 3.1.1. A mean particle size (z-average) of 454 ± 3.2 nm and a PdI of 0.186 ± 0.01 were obtained. The particle size was aligned with the acceptance criteria for the IMP IMIGel (z-average 400 ± 200 nm, PdI <0.3). Other formulation parameters, such as the platform itself, were compared in terms of their *in vitro* and *ex vivo* behavior. Section 3.3 provides a detailed discussion of this comparison.

**FIGURE 3:**
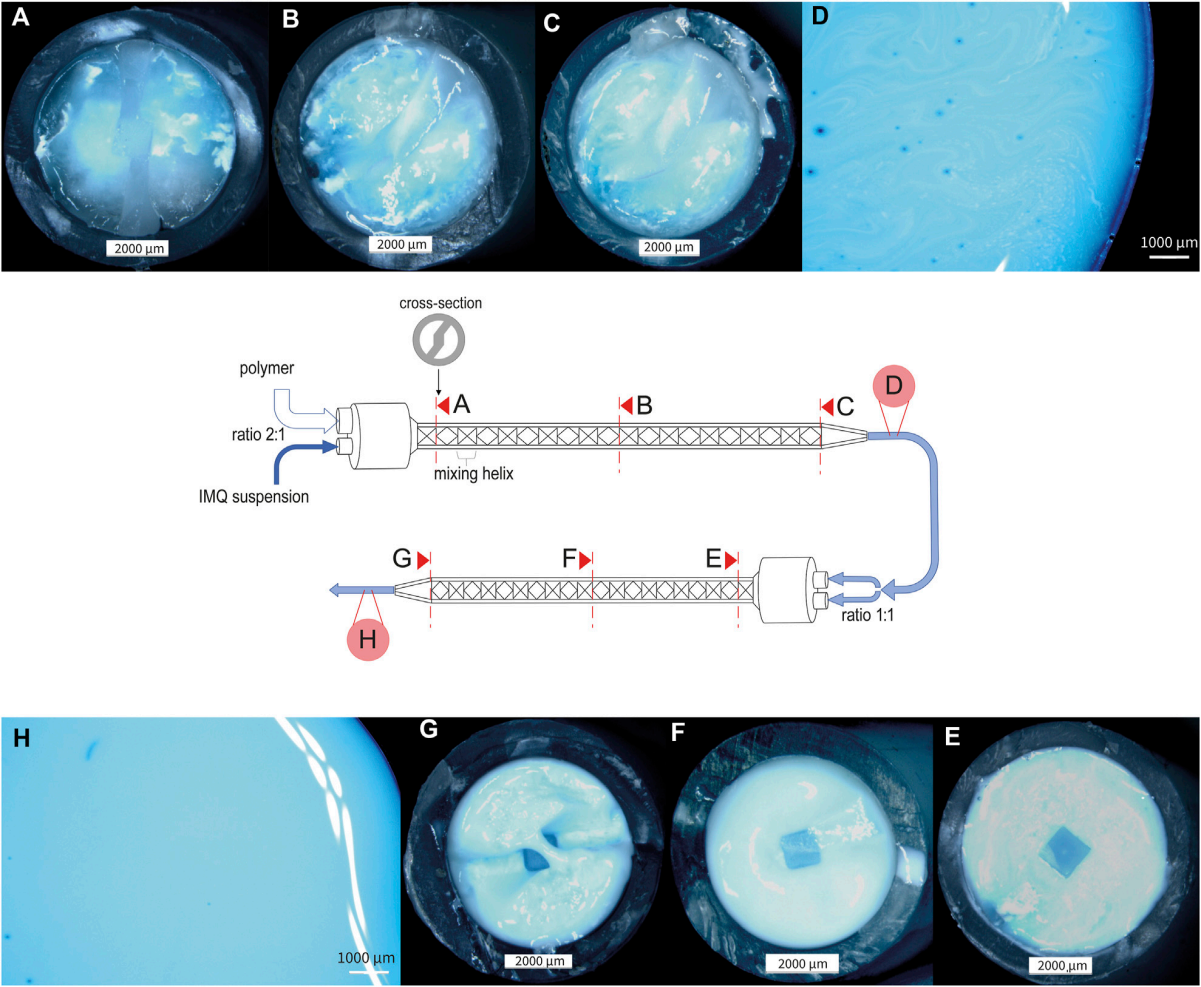
Stereomicroscopic pictures of nanocrystalline IMQ formulation with PVA 18–88 15% (w/w) (1:2) during the mixing process (dyed with methylene blue). **(A)** Cross-section of static mixer MBH 05-20T after the first mixing helix, illustrating the initial stage of the mixing process for the IMQ/PVA formulation; **(B)** Cross-section of static mixer MBH 05-20T after ten mixing helices, showcasing the intermediate stage of the first mixing step **(C)** Cross-section of static mixer MBH 05-20T after twenty mixing helices, representing the conclusion of the first mixing process (similarly mixed as in **(D)**); **(E)** Cross-section of static mixer MBH 06-20T after first mixing helix, illustrating the beginning of the second mixing step; **(F)** Cross-section of static mixer MBH 06-20T after ten mixing helices; **(G)** Cross-section of static mixer MBH 06-20T after twenty mixing helices, signifying the completion of the mixing process [similarly mixed as in **(H)**].

When dealing with the challenge of mixing two fluids with different viscosities, namely, the matrix polymer PVA 18–88 15% (w/w) and the aforementioned IMQ suspension, we evaluated the mixing quality after different mixing procedures. As depicted in Figure 3, progressive improvement in the homogeneity of the mixture was observed, particularly after the second pass through the static mixer. At this stage, the suspension exhibited a distinctly homogeneous appearance (refer to Figures 3G, H). Due to the challenges in visually assessing the homogeneity of the IMQ suspension and the presence of minor inhomogeneities in the final mixture (as shown in Figure 3H), it is essential to conduct further analysis of the samples through HPLC after representative sampling.

**FIGURE 4:**
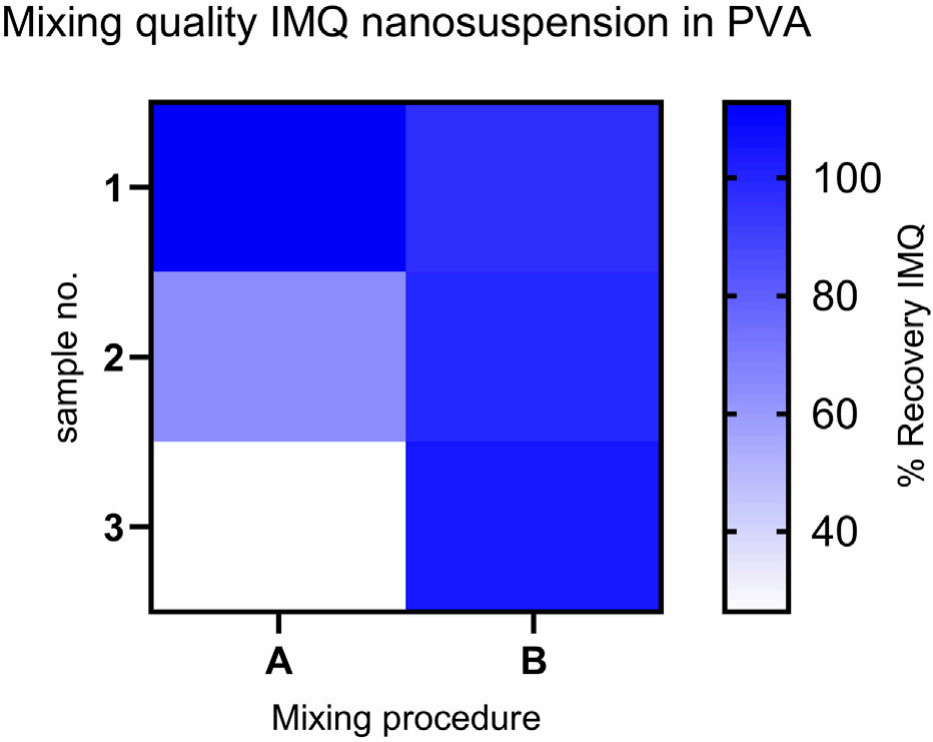
Comparison of IMQ content: Recovery in samples from mixing procedures **(A, B)** (*n* = 3) for the mixed formulation (IMQ nanocrystalline suspension: PVA 18-88 15% (w/w) (1:2)). Content assay conducted by HPLC. (Procedure A: Relative Standard Deviation (RSD) - 52.08%); Procedure B: RSD- 3.51%).

However, we substantiated the significance of our concept for mixing the molding compound by combining these findings with HPLC analysis of the suspension following different mixing steps (see Figure 4). The relative standard deviation (RSD) of the IMQ content in the samples was minimized by switching from mixing procedure A (Static mixer MBH 05-20T 1:2) to mixing procedure B (Static mixer 1:2 + Static mixer MBH 06-20T 1:1), reducing it from 52.08% to 3.51% (refer to Table 2). This ensures uniform drug application in the subsequent manufacturing step of the MNA, resulting in consistent drug loading of the final product.

**TABLE 2:** Evaluation of mixing procedures A and B based on Relative Standard Deviation of IMQ content in samples of the mixed formulation (IMQ nanocrystalline suspension: PVA 18–88 15% (w/w) (1:2)) (*n* = 3).

Mixing procedure		RSD recovery IMQ [%]
A	Static mixer MBH 05-20T 1:2 (20 helix elements	52.08
B	Static mixer 1:2 + Static mixer MBH 06-20T 1:1 (40 helix elements in total	3.51

### 3.2 Characteristics of MNA

#### 3.2.1 Optical appearance and insertion success

**FIGURE 5:**
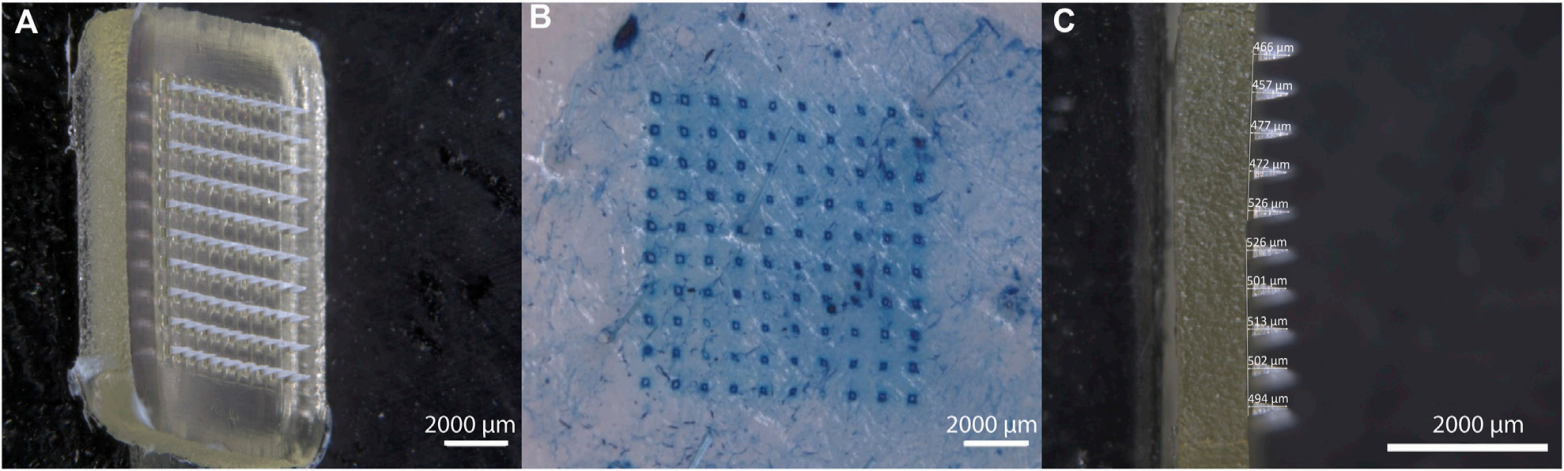
Characterization of IMQ-loaded MNA. **(A)** Stereomicroscopic image (z-stack) of IMQ-loaded MNA before insertion; **(B)** visualization of methylene blue-dyed porcine cadaver ear skin after MNA insertion and removal; **(C)** Lateral view of IMQ-loaded MNA (all Zeiss Discovery V12 stereomicroscope).

Figure 5A depicts a high-resolution view of the IMQ-loaded MNA before insertion into the skin. The z-stack imaging allows for a comprehensive examination of the three-dimensional structure of the MNA. Through this optical analysis, we were able to confirm that the manufactured MNA was homogenous, sharp, and uniform. The tips of the MNA exhibited an average length of 492 ± 28.6 µm (n = 10) (Figure 5C). Only MNAs that appeared to be intact and looked uniform based on visual assessment were selected for further experiments.

After inserting the MNA into porcine cadaver ear skin, the resulting perforation of the upper skin layers was visualized (Figure 5B). Methylene blue dye was applied to highlight the perforation sites, allowing for clear identification and examination of the created microchannels. An insertion success rate of 99.5% ± 0.22% (n = 6 MNA) was observed for the IMQ-loaded MNA, revealing no significant difference compared to the insertion success rate of placebo MNA (99% ± 1.12% (n = 6 MNA)).

Additionally, we were able to prove that epidermal perforation of the porcine skin had been achieved after insertion of the MNA by determining the TEWL before and after insertion. The TEWL significantly increased after insertion, averaging from 16 ± 1.6 g/(m^2^h) (n = 3) to 20 ± 1.2 g/(m^2^h) (*n* = 3).

In conclusion, examining the optical appearance as well as the insertion success of the manufactured MNAs provides essential insights into how the IMQ-loaded MNA can be characterized. This approach enabled us to assess the MNA’s structure and integrity before insertion, as well as to confirm the successful mechanical delivery of the needle tips through the upper skin layers. These visualizations at the nanoscale allow us to better understand the design, performance, and potential applications of MNA for transdermal drug delivery.

#### 3.2.2 Fracture force of PVP backing layer

The fracture force of the polyvinylpyrrolidone (PVP) backing layer of the MNA was determined to be higher than the force exerted by the applicator during MNA insertion into the skin. This indicates that the manufactured MNA assures sufficient platform stability for controlled application.

To measure the force applied by the applicator (Micropoint Technologies Pte, Ltd., CleanTech Loop, Singapore), we utilized a TA. XT plus Texture Analyzer from Stable Micro Systems. The force was measured to be 9.3 ± 0.32 N. In comparison, the fracture force of the manufactured MNA was higher in all tested samples, with a mean value of 38 ± 17.86 N (*n* = 9). It is important to note that the test setup applies force to one point, while the actual application distributes force evenly over the surface of the backing layer. Additionally, we observed a decrease in fracture force when the MNA were exposed to humidity. This finding shows that storing MNAs under low humidity conditions is beneficial for maintaining consistent mechanical properties.

The measurement of the fracture force can also serve as a quality control measure for further scale-up by establishing a minimum threshold for fracture force that should be met.

#### 3.2.3 Drug loading of MNA

The drug loading of the MNA is considered as one of the most critical quality attributes of the formulation. It can be influenced by various process parameters during the manufacturing. These parameters include the mixing procedure of the molding compound (as discussed in Section 3.1.2), centrifugation time, temperature, selection of matrix polymer, and others.

By developing the manufacturing process outlined above, we successfully produced MNA with a drug loading of 70 ± 9.64 µg of IMQ per MNA (n = 11, mean ± SD). This achievement demonstrated our ability to control and optimize the drug loading to meet the desired specifications of the formulation.

### 3.3 *In vitro* comparison of IMQ release

**FIGURE 6:**
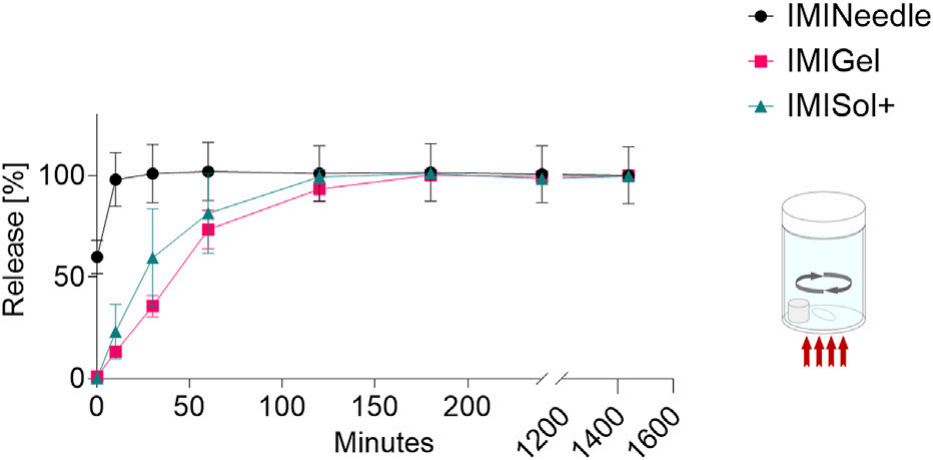
*In vitro* release of IMQ from different formulations: Comparing MNA (dose = 70 µg IMQ) to two semisolid formulations of IMQ (dose = 1,000 µg IMQ) (*n* = 3; data are presented as mean ± SEM).

By comparing the *in vitro* release of the three IMQ formulations, the MNA formulation showed a fast onset with a release of 60% within the first minutes. In contrast, the semisolid formulations showed slower release by exceeding 60% release after 60 min. The data is shown graphically in Figure 6. The fast onset release of the MNA is probably due to the easy accessibility of the solvent to the active ingredient embedded in a dissolvable polymer matrix and close to the surface of the formulation due to the geometries of the MNA. However, the data on the correlation between *in vitro* and *in vivo* release of MNA is poor. To address this issue, we further conducted experiments on the impact of the application time and *ex vivo* permeation data (described in sections 3.4 and 3.5).

### 3.4 *Ex vivo* comparison of IMQ permeation

**FIGURE 7:**
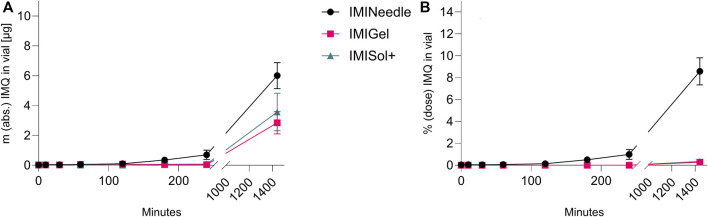
*Ex vivo* permeation plot of IMQ across porcine cadaver ear skin using Franz diffusion cell (*n* = 6) over 24 h: Comparing MNA (dose = 70 µg IMQ) to two semisolid formulations of IMQ (dose = 1,000 µg IMQ). **(A)** Absolute Permeation of IMQ through the skin; **(B)** Permeation relative to the applied dose comparison (data are presented as mean ± SEM).

By comparing the two semisolid formulations, IMIGel and IMISol+, with the manufactured MNA (IMINeedle), we can assess the absolute and relative *ex vivo* permeation of IMQ through porcine cadaver ear skin. The experimental data are presented in Figure 7, where each point represents the mean of six replicates along with the standard error of the mean. Additionally, the data is summarized in Table 3.

**TABLE 3:** Results of Franz diffusion cell *ex vivo* permeation testing after 24 h of the manufactured formulations (Means ± SEM).

Formulation	IMQ dose applied [µg]	Permeation IMQ % dose	Permeation IMQ abs. [µg]
IMINeedle	70	9 ± 1.13	6.0 ± 0.79
IMIGel	1,000	0.28 ± 0.07	2.9 ± 0.68
IMISol+	1,000	0.4 ± 0.10	4 ± 1.03

**FIGURE 8:**
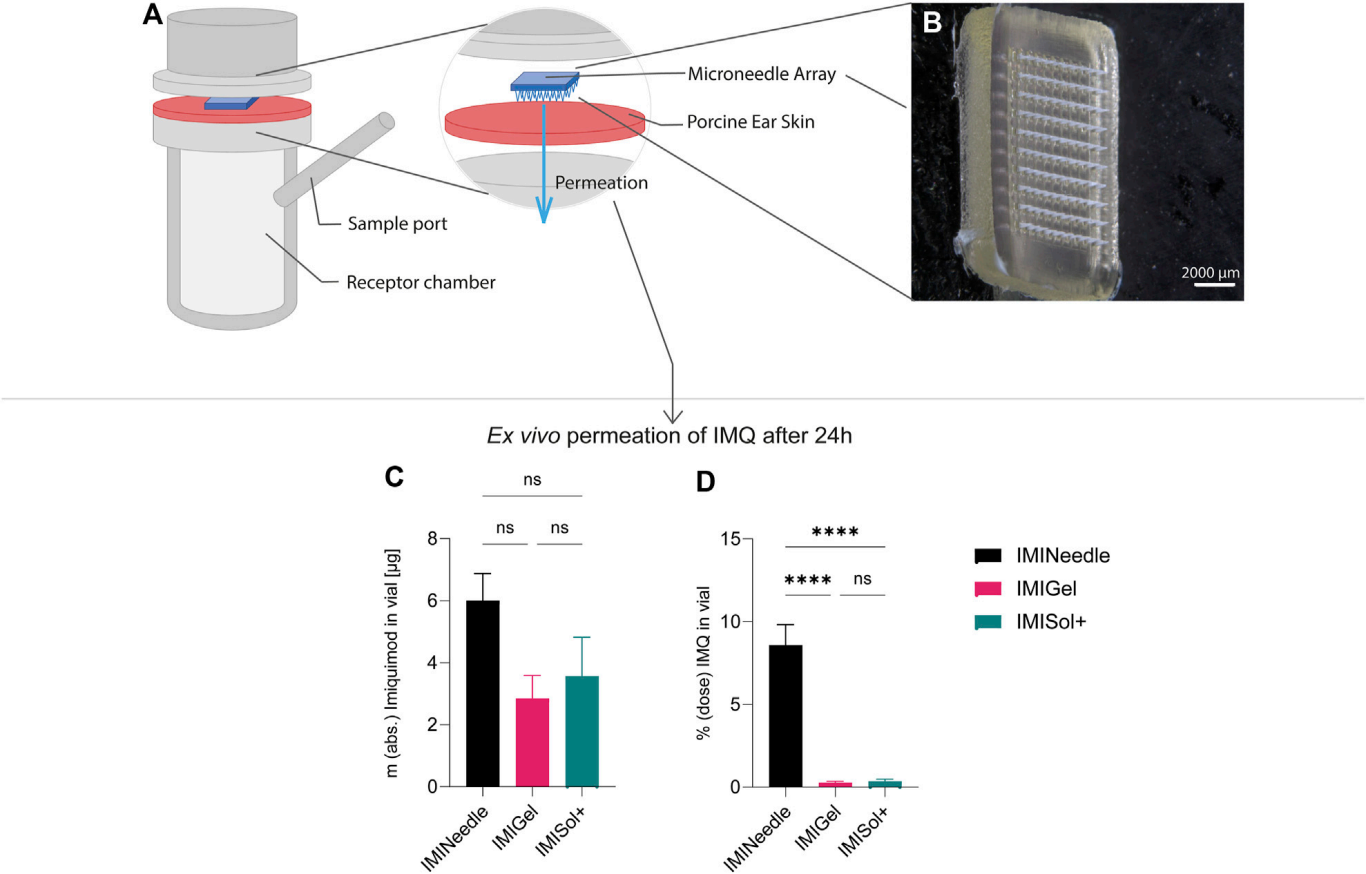
Summary of *ex vivo* permeation data of IMQ across porcine cadaver ear skin using Franz diffusion cell (n = 6) over 24 h: Comparing MNA (dose = 70 µg IMQ) to two semisolid formulations of IMQ, showing means ± SEM (ns, *p* > 0.05; ****, *p* < 0.0001). **(A)** Schematic setup of Franz diffusion cell. **(B)** Stereomicroscopical image of representative MNA used in *ex vivo* experiments. **(C)** Absolute *ex vivo* permeation of IMQ after 24 h. **(D)** *Ex vivo* permeation of IMQ after 24 h relative to the dose applied.

Furthermore, a one-way ANOVA followed by Tukey’s *post hoc* analysis revealed that *ex vivo* permeation of IMQ significantly differed (*p* < 0.001) between IMINeedle and the semisolid formulations relative to the applied dose (Figure 7B). The mean permeation (% dose) decreased from IMINeedle to IMIGel (8.30, 95%-CI [5.54, 11.06]) and from IMINeedle to IMISol+ (8.23, 95%-CI [5.33, 11.12]) while showing no significant difference between IMIGel and IMISol+ (−0.07, 95%-CI [-2.97, 2.82]) as depicted in Figure 8. These findings affirm that IMQ delivery via MNA is more efficient compared to topically applied semisolid formulations.

In addition, the analysis of the results showed no significant difference in the absolute *ex vivo* permeation of IMQ between formulations. This demonstrates that the applied doses of IMQ were equivalent and comparable. The approach we chose may enable us to further substantiate the beneficial characteristics of MNA as a platform for transcutaneous drug delivery.

### 3.5 *Ex vivo* comparison of IMQ permeation upon different application durations

**FIGURE 9:**
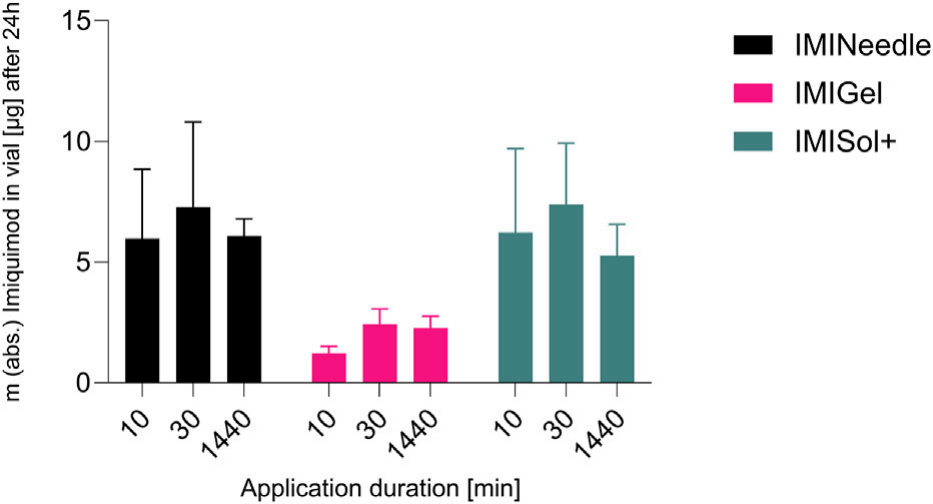
Comparison of application durations of different IMQ formulations: Summary of *ex vivo* permeation data of IMQ across porcine cadaver ear skin using Franz diffusion cell (*n* = 3) over 24 h after application of 10, 30, and 1,440 min: Comparing MNA (IMINeedle; dose = 70 µg IMQ) to two semisolid formulations of IMQ (IMIGel, IMISol+), showing means ± SEM.

The study examined the application time of the formulations, as their *in vitro* release differed in the initial period of release testing. Interestingly, no significant impact on the percentage of dose permeated *ex vivo* after 24 h was observed despite varying application times as shown in Figure 9. Deviations from the expected results as the decrease in permeation after 24 h of application in all formulations could be attributed to the use of bio-membranes which cannot provide consistent qualities over all batches. However, the relative *ex vivo* permeation of IMQ (related to the applied dose of 70 µg respectively 1,000 µg IMQ) was highest in the MNA formulation. These findings suggest that the formulations, particularly the MNAs, do not require extended application times to deliver the drug IMQ. This is advantageous for patient application, as it reduces the potential for misapplication when the duration of formulation attachment to the skin is reduced.

### 3.6 *In vivo* effect of IMQ in MNA

**FIGURE 10:**
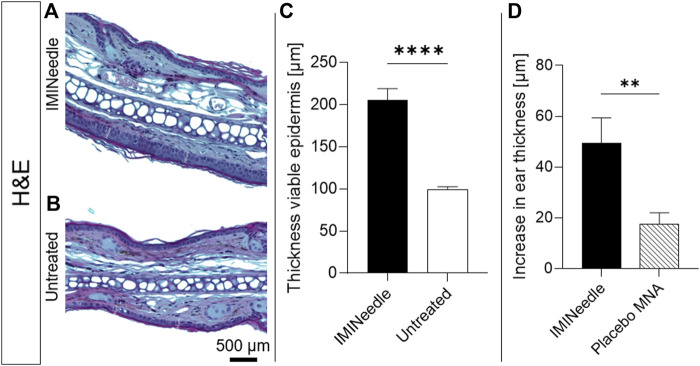
Effects of IMINeedle on mouse ears. Representative microscopical images after H&E staining of **(A)** IMINeedle treated ear skin and **(B)** untreated ear skin. The scale bar on **(B)** can be applied to both images. **(C)** Evaluation of thickness of the viable epidermis on day 7 (*n* = 9, data showing mean ± SEM; ****, *p* < 0.0001). **(D)** Increase in ear thickness with treatment IMQ-loaded and Placebo MNA 2 days after application (n = 10, data showing mean ± SEM; **, *p* < 0.01).

The study utilized the IMQ-induced swelling of the viable epidermis to evaluate the effectiveness of the MNA formulation. Figures 10A, B show representative microscopic images of H&E-stained mouse ears on the seventh day after the application of the MNA and untreated mouse ears, respectively. The results showed a significant increase in epidermal thickness to 206 ± 13 µm in IMINeedle-treated mouse ear skin, compared to the untreated control group (Figure 10C). To ensure that the swelling of the mouse ears is not only induced through the puncturing by the MNA, the total ear thickness 2 days after application of IMQ loaded MNA as well as placebo MNAs was measured. The results showed a significant increase in ear thickness of the IMINeedle-treated mouse ears of 50 ± 9.9 µm, which supports that the swelling is caused by the applied drug, IMQ (Figure 10D).

## 4 Discussion

The study results provide a comparison of the performance of dissolving MNA with previously developed semisolid formulations as dermal delivery systems for nanocrystalline IMQ. To enhance the performance of the formulation of IMQ from semisolid to MNA, it is highly suitable to maintain the particle size in the upper nanometer scale, especially in the context of transcutaneous immunization (Gogoll et al., 2016). Previous studies have reported on the advantages of MNA containing dissolved IMQ over semisolid formulations (Sabri et al., 2020; Chiu et al., 2021). Additionally, microneedle pretreatment has been shown to enhance skin permeation (Al-Mayahy et al., 2019). In our study, we formulated IMQ as nanocrystals and combined them with the unique features of the MNA platform. This combination has led to an improvement in drug delivery efficacy, doubling its effectiveness compared to the system used by Sabri et al. (2020). They employed a fast-dissolving MNA incorporating HCl-dissolved IMQ and compared it to the semisolid IMQ formulation Aldara™ (Sabri et al., 2020). Overall, the MNA platform is advantageous for delivering nanoscale particles, as reported in studies demonstrating favorable characteristics (Sharma et al., 2019; Park et al., 2021; Petrová et al., 2024).

Expanding on the concept of dermal application of nanocrystalline IMQ, the manufacturing of dissolvable MNAs using polyvinyl alcohol (PVA) and polyvinylpyrrolidone (PVP) backing has yielded promising results in terms of optical, mechanical, and drug-loading properties.

Throughout our studies, we defined basic product requirements to facilitate the formulation of nanocrystalline IMQ for topical administration. We decided on microneedles as a promising platform for the formulation, and defined a first quality target product profile and critical quality attributes based on the studies with the semisolid formulation IMIGel (Pielenhofer et al., 2023). Besides the target properties of the formulation itself, we had to focus on feasibility for the manufacturing process. For this, we screened different polymers to be used as a matrix for the microneedles (e.g., PVA with different molecular weight and degree of hydrolysis) which should combine the properties of water solubility, suitable viscosity in solution >15% (concentration was set to this level to keep the drying loss after molding at an acceptable level) and biocompatibility. The viscosity of the polymer solution was important for the mixing process, the centrifugation step as well as the pipetting into the molding templates. As surrogate parameter for “good” viscosity, we checked the resulting formulations for drug loading and homogeneity within the manufactured batch by using HPLC.

After manufacturing microneedles with the screened polymers, they were optically checked, whether they could be successfully demolded from the templates. For this the geometries of the needle tips as well as their length was determined. The promising candidates were then checked for their insertion capabilities as well as their mechanical stability.

As a result, we defined the reproducible insertion success as well as reproducible drug loading and release capacities as basic features of the microneedles we wanted to formulate. To ensure these properties, we conducted the tests described throughout the manuscript. In particular, we observed high insertion success of the microneedle tips (>99%) and significant increase in TEWL after insertion, when using microneedles which were optically uniform and intact. This resulted in the assumption, that we could rely on the insertion properties when adhering to the defined manufacturing process. As the reproducible application force of the microneedles is ensured by using an applicator, the mechanical stability of the backing layer supporting the microneedle tips for insertion should withstand the applied force, which was defined as a force limit of >9 N. The uniformity in drug loading of the microneedles was ensured by the described mixing and molding process, which was controlled for representative samples using HPLC. Here, we first defined a limit of 15% deviation from the mean, which we were able to fulfill. However, the process should be further developed to achieve regulatory compliance to continue the development of an adequate drug product. This process has also revealed potential aspects for quality control in scale-up processes. The production of MNA can be significantly streamlined by employing a cost-effective, gentle, and sterilizable mixing method involving static mixing elements. This not only paves the way for the production of high-quality MNA incorporating nanocrystalline IMQ but also presents opportunities for other suspension-based formulation approaches.

The most important results of the investigations are the conclusions we draw from the combination of the release and permeation data. Here we see an initially rapid *in vitro* release of the active ingredient from the MNA formulation, which exceeds the release compared to the semi-solid formulations. This rapid influx is not reflected in the *ex vivo* permeation of the same formulation. It is reasonable to assume that the release of IMQ from the MNA is initially rapid, but that the permeation over the full-thickness porcine ear skin is then less dependent on the initial release. This assumption is supported by the data from the study on the application time of the formulations. Even after only a short application time of less than 1 hour, similar *ex vivo* permeation data can be obtained after 24-h application of the same formulations. This effect of the application time on the *ex vivo* permeation is also evident in the semi-solid preparations investigated, which leads to the assumption that the permeation of the nanocrystalline IMQ is only conditionally dependent on the formulation. In conclusion, the dissolution of the investigated formulations seems to be prior to the diffusion and permeation of IMQ through the skin.

Despite this independence, there are clear advantages of the MNA formulation compared to the semi-solid preparations, which are mainly reflected in the simplicity of application, the reproducibility of the dose on the patient (especially compared to semi-solid preparations), and the reduction of the dose to a 15th of that used in the investigated semisolid formulations and thus increase in efficacy. Unlike patients who need to estimate the appropriate amount of semisolid formulation to apply, MNAs provide a standardized approach with a defined contact duration, ensuring consistent coverage of the treated area when the MNA patch adheres to the skin. These findings also suggest the potential to improve patient compliance by reducing the incidence of topical side effects associated with IMQ, which has a broad spectrum of adverse reactions (Cantisani et al., 2012). However, further experiments are needed to validate this suggestion.

## Data Availability

The raw data supporting the conclusion of this article will be made available by the authors, without undue reservation.
